# Simulation Modeling as a Novel and Promising Strategy for Improving Success Rates With Research Funding Applications: A Constructive Thought Experiment

**DOI:** 10.2196/18983

**Published:** 2020-07-30

**Authors:** Allen McLean, Wade McDonald, Donna Goodridge

**Affiliations:** 1 College of Medicine University of Saskatchewan Saskatoon, SK Canada; 2 Department of Computer Science University of Saskatchewan Saskatoon, SK Canada

**Keywords:** simulation modeling, computational science, funding application, grant funding, grant writing, nursing, research, thought experiment, persuasive technology, peripheral vascular disease

## Abstract

Writing a successful grant or other funding applications is a requirement for continued employment, promotion, and tenure among nursing faculty and researchers. Writing successful applications is a challenging task, with often uncertain results. The inability to secure funding not only threatens the ability of nurse researchers to conduct relevant health care research but may also negatively impact their career trajectories. Many individuals and organizations have offered advice for improving success with funding applications. While helpful, those recommendations are common knowledge and simply form the basis of any well-considered, well-formulated, and well-written application. For nurse researchers interested in taking advantage of innovative computational methods and leading-edge analytical techniques, we propose adding the results from computer-based simulation modeling experiments to funding applications. By first conducting a research study in a virtual space, nurse researchers can refine their study design, test various assumptions, conduct experiments, and better determine which elements, variables, and parameters are necessary to answer their research question. In short, simulation modeling is a learning tool, and the modeling process helps nurse researchers gain additional insights that can be applied in their real-world research and used to strengthen funding applications. Simulation modeling is well-suited for answering quantitative research questions. Still, the design of these models can benefit significantly from the addition of qualitative data and can be helpful when simulating the results of mixed methods studies. We believe this is a promising strategy for improving success rates with funding applications, especially among nurse researchers interested in contributing new knowledge supporting the paradigm shift in nursing resulting from advances in computational science and information technology.

## Introduction

Establishing a successful career as a nurse researcher working in a faculty or research position depends on a variety of factors. These factors typically include contributions toward research and publishing, teaching, service, and clinical practice [1], among others. Mastering each of these demanding roles is essential for retention, promotion, and tenure [2,3]. As part of their role in research, nurse researchers must write grant proposals and other funding applications to support their work, a challenging task [4,5], with often uncertain results. Recent (2014-16) success rates in the Canadian Institute of Health Research open grant competitions have hovered around 12% [6]. In similar competitions and over a similar period, the National Institute of Health (USA), Australian Research Council, and Medical Research Council (United Kingdom) reported better (but still low) success rates around 18%, 20%, and 24%, respectively [7-9]. These low success rates not only threaten the ability of nurse researchers to conduct relevant health care research [10] but may also negatively impact their career trajectories.

Grant writing is an established method for supporting research, enhancing institutional prestige, and promoting individual career advancement [11,12]. Many individuals and organizations have offered advice for improving success with funding applications [11-15]. Their recommendations often overlap, with consistent themes emerging—nicely summarized by Wisdom, Riley, and Myers [15]:

(1) research and identify appropriate funding opportunities; (2) use key proposal components to persuade reviewers of project significance and feasibility; (3) describe proposed activities and their significance persuasively, clearly, and concisely; (4) seek review and feedback from colleagues; (5) establish a study design that is simple, logical, feasible, and appropriate for the research questions; (6) develop a timeline for the proposal process; (7) select a novel, high-impact project; (8) conduct an exhaustive literature review; (9) ensure that budgets are reasonable; and (10) consider interdisciplinary collaborations.

And while these are all excellent recommendations, they are also common knowledge and simply form the basis of any well-considered, well-formulated, and well-written funding application. For nurse researchers interested in taking advantage of innovative methods and leading-edge analytical techniques that could be used to complement the usual recommendations [15], we propose adding the results from computer-based simulation modeling experiments to funding applications. We believe this is a promising strategy for improving success rates with such applications, especially among nurse researchers interested in contributing new knowledge supporting the paradigm shift in nursing resulting from advances in computational science, information technology, and health information science.

This paper is meant to be the first step in a multi-stage strategy intending to test this approach for improving success rates with research funding applications. Initially, we hope to gather feedback from interested individuals that will help us develop a proof-of-concept funding application model that includes simulation modeling experiments. We will then circulate this example for review among individuals with experience assessing funding applications. Based on their feedback, we will either: (1) discard this concept as either infeasible or ill-advised, (2) rework this concept and recirculate among our experts for further review, or (3) submit a bona fide funding application that includes results from simulation modeling experiments.

## Objectives

This paper is written as a constructive thought experiment to encourage discussion, reflection, and critique. We will briefly introduce some fundamental concepts concerning thought experiments and computer-based simulation modeling—including its utility and limitations (within the context of improving success rates with funding applications), and advice for those interested in exploring this approach further. We will not review the practical details of constructing, calibrating, testing, validating, and reporting credible simulation models in this short paper; appropriate references are provided.

We include an example of a simulated randomized controlled trial (RCT)—a simulation based on an imagined research proposal with multiple experiments describing the use of persuasive health technologies for improving health outcomes among a cohort of people living with peripheral vascular disease. We will demonstrate how the results from these simulation experiments may be used by nurse researchers in a research funding application, and present arguments supporting this approach for improving application success rates.

## Thought Experiments

A thought experiment is an experiment performed in the imagination [16]. Though abstractions, thought experiments are more than ‘just thinking about something,’ and more than a ‘think piece,’ which is defined as a piece of writing meant to be thought-provoking and speculative [17]. Instead, a thought experiment is a deliberate and systematic approach to exploring some problem or idea [18]. Thought experiments are used for hypothesizing, theory selection, theory implementation, conceptual analysis, counterfactual thinking, exploration, education, entertainment, and the opportunity to ask a variety of ‘what if’ questions [18], which are only limited by our curiosity and creativity.

While the value of thought experiments is debated by some [19], it is widely agreed they play an essential role in many disciplines, predominantly physics and philosophy [16]. Well-known examples remind us of their tremendous influence and consequence: Newton’s bucket, Maxwell’s demon, Einstein’s elevator, Leibniz’s mill, Thomson’s violinist, Heisenberg’s gamma-ray microscope, and Schrodinger’s cat [18-20], to name a few. Thought experiments played a crucial role in the development of quantum mechanics and Einstein’s theory of relativity [18].

The modest thought experiment presented in this paper is based on a taxonomy proposed by Brown [21], the *constructive thought experiment*. Constructive (also known as apologetic or heuristic) thought experiments aim to provide positive support for an idea, concept, or theory and are often developed as a heuristic aid, enabling a person to discover or learn something new for themselves [21]. A constructive thought experiment is an appropriate framework with which to investigate the promise of simulation modeling for improving success with research funding applications; the approach is novel and promising, as yet untested by the authors, and we are unable to find either published or anecdotal evidence for same.

## Simulation Modeling

Simulation modeling is a systems science and computational methodology that examines behaviors and outcomes resulting from interactions, linearities/nonlinearities, and feedback loops occurring between multiple system actors over time [22]. Simulation modeling is a robust research methodology for theory development, testing, critique, and refinement. Simulation modeling is a rich, robust, and versatile research tool—dynamic, highly visual, and on the leading edge of health care research [23,24]. As with thought experiments, simulation modeling allows nurse researchers the opportunity to ask a variety of ‘what if’ questions using a deliberate and systematic approach. Simulation modeling can be viewed as a method for *operationalizing* a thought experiment, allowing nurse researchers the opportunity to present their understanding of a system or problem in a tangible form that can be more readily shared and scrutinized.

Experts in nursing science view advanced computational techniques as necessary for moving nursing research, policy, education, and practice into the future [25,26]. Conveniently, the broad concept of simulation will not be mysterious or unfamiliar to most nurses. Role-play simulations are simplified and safe reproductions of real-world health care situations [27]. High-fidelity simulation manikins have been used effectively for many years to teach clinical skills [28], and nursing simulations using virtual or augmented reality are now proving valuable in clinical education [29,30], and practice [31]. Computer-based simulation modeling creates a unique type of simulation experience. Using simulation software, nurse researchers can build a virtual space where they may create models of real-world systems, and then explore a wide array of research questions within those computer-based worlds. These models produce theoretical outputs based on modifiable input data, giving nurse researchers the ability to examine the behaviors of complex systems over a wide range of hypotheses [32]. A computational simulation strategy is cost-efficient, but it also allows nurse researchers to explore problems in a risk-free environment where they can experiment, make mistakes, refine their models, assumptions, or interventions, and begin again [33].

There are three types of simulation modeling approaches commonly used; system dynamics, agent-based, and discrete-event (also termed process modeling). It is also possible to combine these approaches and produce hybrid (also termed multi-method) models. Only a synopsis of each simulation modeling approach will be presented in this brief paper, but references are included for those readers interested in delving deeper into the subject.

### System Dynamics

System dynamics (SD) is a highly abstract method of modeling. This approach typically ignores the fine details of a system and produces a high-level representation of a system or problem. These simulation models are often used for long-term planning and strategic decision-making [34]. SD simulation modeling has been used in epidemiologic research and policy planning for many years and continues to provide new insights [35]. Interested readers are encouraged to review papers describing SD simulation modeling: in greater detail [34], with applications in health care [36], and health care policy [37].

### Agent-Based

Agent-based modeling (ABM) simulates the characteristics, behaviors, and interactions between autonomous agents and their environment [38]. Agents may be any entity we wish to represent, anything from the real world important for answering a research question. We can give our agents attributes, define the behaviors of those agents, place those agents in a simulated environment, establish connections and relationships between agents—then create scenarios, and run experiments. Nurse researchers can then observe the global behavior of their model over time, resulting from the many interactions of the individual agents and environment [39]. Interested readers are encouraged to review papers and texts describing agent-based simulation modeling: in greater detail [34,36,40,41], with applications in nursing research [26], and health care practice [42,43].

### Discrete-Event

Discrete-event simulation (DES) modeling focuses on the processes in a system at an individual level of abstraction. Many health care processes and problems can be described as a sequence of separate, discrete events. DES is particularly useful for modeling resource-constrained workflows, such as patient flow through an outpatient clinic, perioperative suites, or emergency department. DES modeling is widely used in health care, and interested readers are encouraged to review papers describing discrete-event simulation modeling: in greater detail [34,36], and with applications in health care [44,45].

## Hypothetical Example

The purpose of the hypothetical example we present here is to demonstrate how the results of simulation modeling experiments could be useful and advantageous when included with a typical funding application. This example is fictitious and is not meant to represent an actual or complete funding application; as such, much of the detail and referencing is unnecessary and is not included. Naturally, our example presents positive results, and for obvious reasons, we would advise nurse researchers against including the results of simulation modeling experiments in funding applications, which indicated otherwise. Our example describes a hypothetical RCT—a simulation based on an imagined research proposal with multiple experiments describing the use of persuasive health technologies for improving health outcomes among a cohort of people living with peripheral vascular disease.

### Background

Chronic diseases are among the most common, costly, and preventable of all health problems worldwide. In 2016, roughly 244,000 (89%) of the 273,000 deaths registered in Canada were attributable to chronic diseases. Peripheral vascular disease (PVD) is a serious chronic health problem affecting blood vessels throughout the body, excluding the heart and brain. PVD interferes with normal circulation, and the long-term sequelae include a higher susceptibility to lower-limb and foot wounds—serious wounds frequently leading to infection, ulceration, gangrene, and ultimately surgical amputation. Persuasive health technologies (eg, eHealth programs, mHealth apps) typically use theories of motivation and persuasion to influence, reinforce, change, or shape health-related attitudes and behaviors.

### Objective

The study aim is to investigate the efficacy of a behavioral intervention delivered using persuasive health technologies (smartphone app) to align study participants’ lower-limb self-care behaviors with clinical guidelines, among people living with PVD. Aligning self-care behaviors with clinical guidelines assists with the earlier identification of changes in the lower-limb and foot that may indicate the early development of serious wounds. With prompt care-seeking and treatment, we can reduce the incidence of complicated lower-limb and foot infections, ulceration, gangrene, and surgical amputation.

### Design

The study is an RCT design, recruiting people living with PVD from centers across western Canada. The goal is to recruit 100 participants from each center, for a total of 700 study participants. Approximately half will be randomized to the control group, with the remaining participants assigned to the intervention group. A variety of metrics (e.g., incidence, resource utilization, costs) will be tracked. The study will be run over 4 years.

### Model

**Figure 1:**
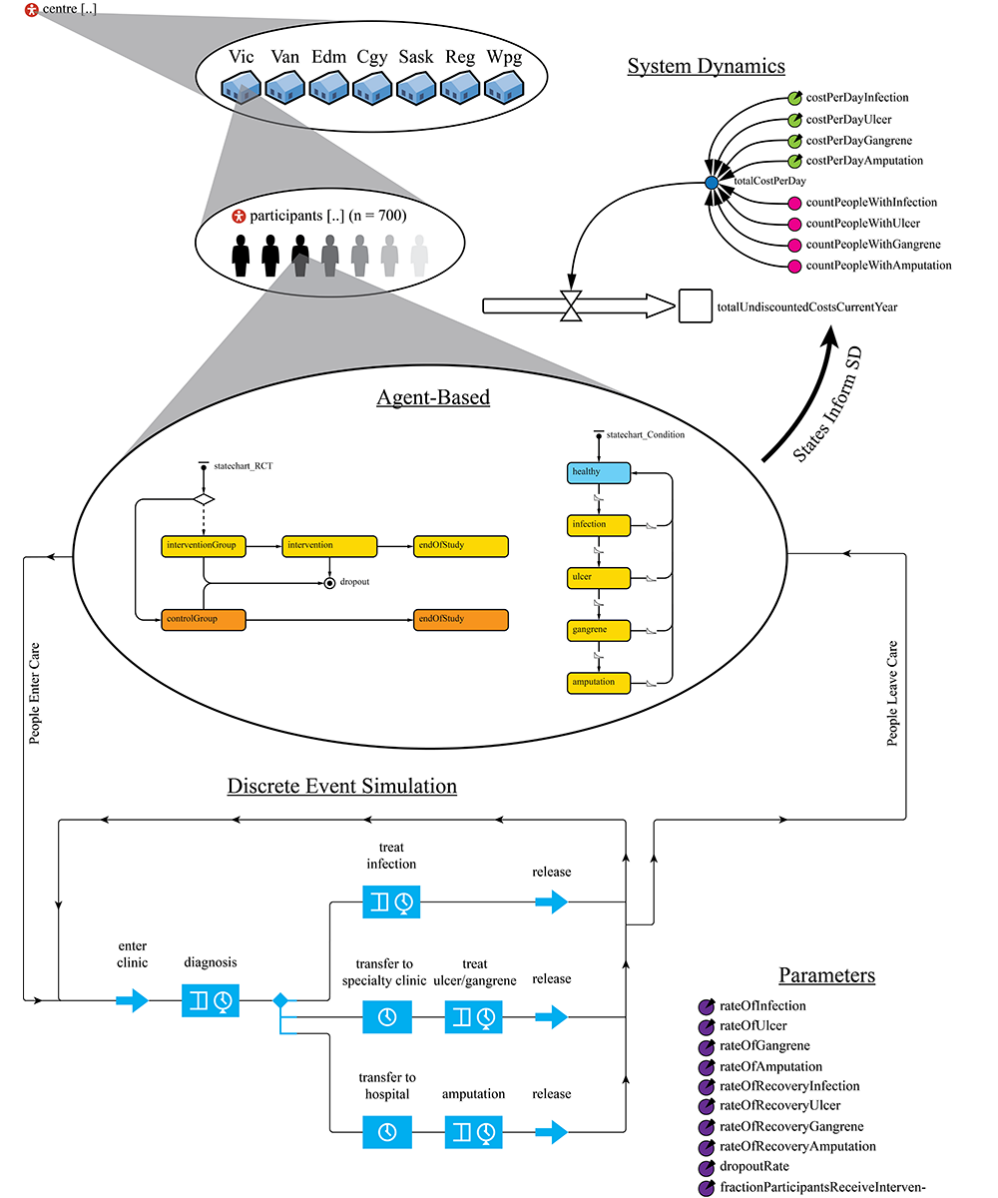
Simulation model design.

A hybrid model was developed using elements from system dynamics, agent-based, and discrete-event simulation modeling (Figure 1). Each element and their associated connections represent some entity or relationship from the real world necessary for answering our research objective. Figure 1 is an example of a typical user interface a nurse researcher might see on their computer screen when building a model using simulation modeling software; in this case, AnyLogic simulation software [46]. One of the key benefits of using simulation modeling over some other modeling approaches (eg, Excel), is its ability to provide a visual representation of the research design, which is helpful when explaining a simulation modeling approach to people unfamiliar with the concept.

The ABM component in Figure 1 depicts the recruitment of participants from each of the seven centers, randomization between control and intervention arms (gold and orange rectangles), and the possible health states of each fictional study participant, ranging from healthy to requiring amputation. At any time, an individual health state (blue and red rectangles) is determined by an algorithm, based on the available evidence (eg, incidence and prevalence data, rates of disease progression). For example, an agent might move from the ‘healthy’ state to the ‘infection’ state based on the probability of developing an infection derived from research among people in this population. Also, an agent might move from the ‘ulcer’ state and return to the ‘healthy’ state based on known rates of recovery and informed by treatment assumptions included in the development of this model.

Simulation models must generate sensible results; therefore, the evidence used to create model algorithms must be the same as the evidence used to support the written funding application. It would be especially misleading to create model algorithms designed solely to generate positive results, and we would strongly recommend against such deceit. We recommend nurse researchers provide a transparent description of all assumptions, parameters, and variables guiding their models.

The DES component in Figure 1 illustrates the flow of each fictional study participant through different services within the health care system. In our example, study participants visit primary care for treating infections, specialty clinics for treating ulcers and gangrene, and hospital services providing amputation. Patient flow through these services would be determined by an algorithm, again based on the available evidence (e.g., rates of infection, ulceration, gangrene, amputation, rates of recovery). If a participant remains in a healthy state throughout the study, they will not enter any of the DES care pathways. Resource utilization can be easily tracked within the DES component of the model; for example, cumulative nursing hours spent treating ulcers. If a simulation model demonstrates a reduction in these hours among participants in the intervention arm, this statistic could be added to the other arguments supporting an application seeking funding for that intervention.

The SD component in Figure 1 tracks expected annual costs to the health care system within our hypothesized study. Costs are accumulated using an algorithm based on available cost data for each type of treatment or procedure, and population estimates could be derived after extrapolation. It is essential to understand that each element in this model can ‘communicate’ with other elements. For example, data generated in the DES care pathways (e.g., RN hours spent on each treatment, number of procedures) can be used to inform the cost calculations in the SD component using specific parameters or variables (Figure 1).

### Results

**Figure 2:**
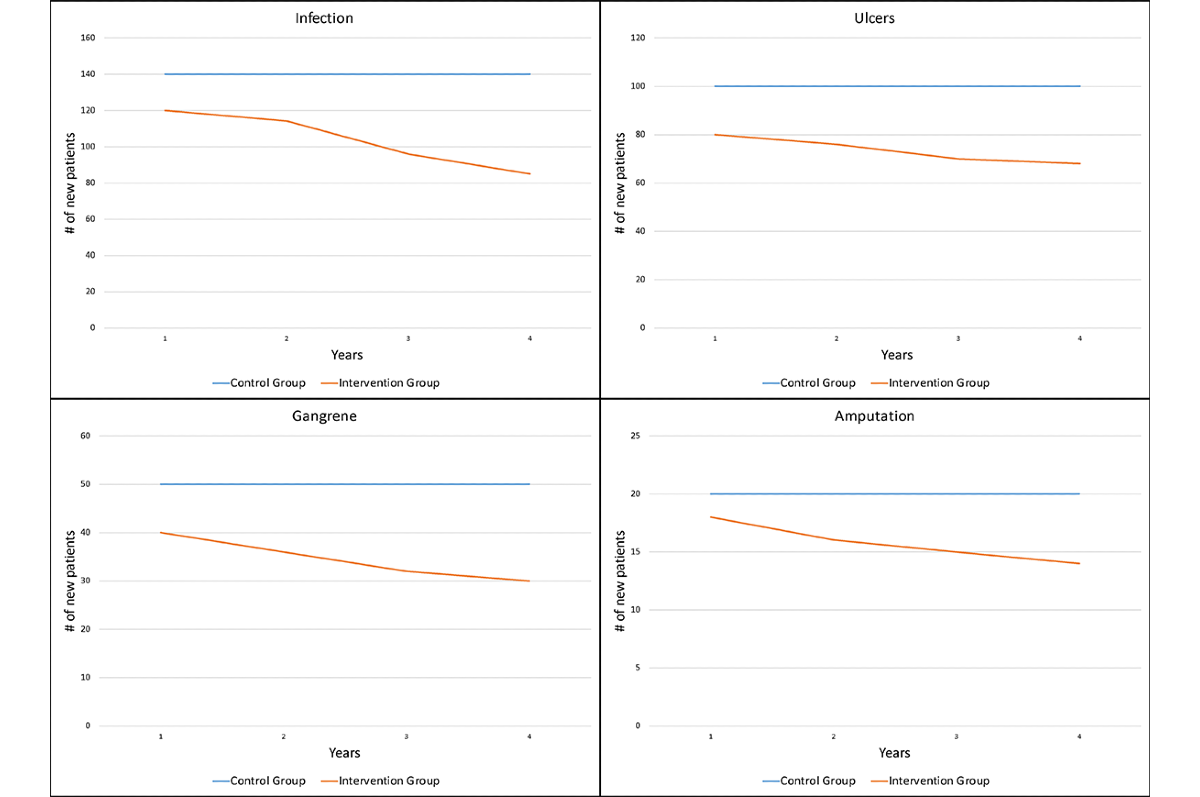
Incidence, per year.

**Figure 3:**
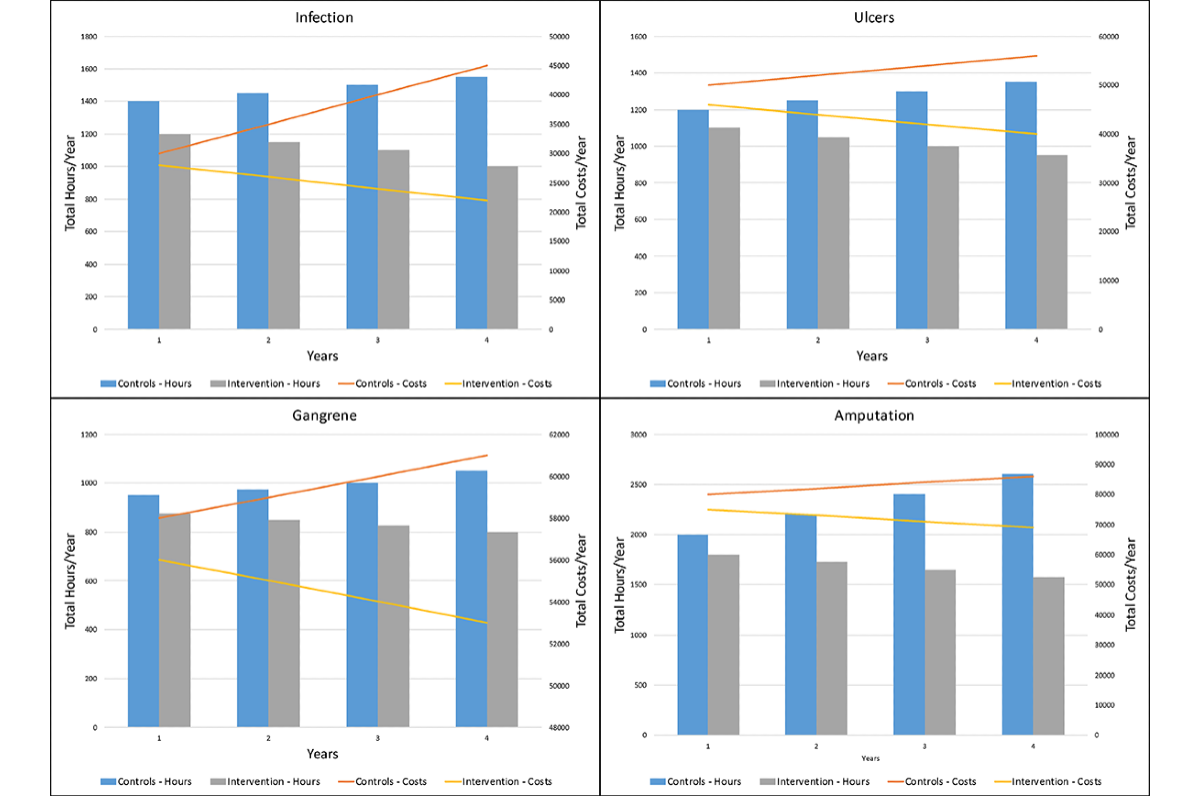
Resource utilization and costs, per year.

Figures 2 and 3 represent only a few examples of the many possible results and output graphs that could be generated by simulation experiments, and that could be included with funding applications. Figure 2 displays the results of our imagined intervention on incidence per year, and Figure 3 displays the results of our intervention on resource utilization and costs per year. And while these results are wholly theoretical and highly dependent on the assumptions and data informing our model, at a minimum, these results can suggest the possibility of positive outcomes in a real-world trial, and proper use of the requested funding.

## Challenges

We anticipate several problems with using the results from computer-based simulation modeling experiments as a strategy for improving success rates with funding applications. Most reviewers will likely be unfamiliar with computer-based simulation modeling, meaning that an adequate explanation of simulation modeling, a description of the model, and results from the simulation experiments must be included with an application. However, typical research funding applications often have strict page restrictions, and there simply may not be enough space to include the necessary background information. Additionally, learning how to use simulation modeling software and creating models will require a serious commitment by interested nurse researchers. Fortunately, educational information is readily available, as are many powerful open-source and proprietary simulation software packages. Most modern computers and laptops are capable of running simulation software and partnering with experienced simulation modelers is an option.

## Conclusion

Nurse researchers face a difficult problem. The competition for scarce research funding is intense, while at the same time, research funding awards are essential for continued employment, promotion, and tenure. Adding to the challenge, nurse researchers must compete among themselves for this funding, and often against researchers from other health care disciplines, undoubtedly limiting the amount of nursing-specific research they can contribute. However, new strategies such as computer-based simulation modeling may prove useful for improving success rates with funding applications. With this paper, we hope to open a discussion among our nursing colleagues about this novel and promising approach. As a next step, we hope to gather feedback from interested individuals that will help us develop a proof-of-concept funding application model that includes simulation modeling experiments.

The World Health Organization has designated 2020 as the International Year of the Nurse and the Midwife, and the International Council of Nurses has chosen the theme [47], Nurses: A Voice to Lead – Nursing the World to Health. *Nursing Now* is a three-year campaign to raise the status and profile of our profession, and nurse scholars, practice leaders, and educators around the world will be working toward influencing and enacting public and health policy globally, from a nursing perspective [48]. As nurse researchers and colleagues, we can contribute valuable insights toward these goals through our research; securing additional funding will result in additional research and knowledge we can translate from a nursing perspective. Surely this makes computer-based simulation modeling a strategy worthy of discussion and consideration.

## Abbreviations

ABM: agent-based modeling
DES: discrete-event simulation modeling
PVD: peripheral vascular disease
RN: registered nurse
SD: system dynamics modeling

## References

[1] Chase Jo-Ana D, Thiele Doria K (2015). Continuing the Journey: Transitioning to the New Tenure-Track Faculty Role. West J Nurs Res.

[2] Singh MD, Patrick L, Pilkington B (2016). An Exploration of the Pre-Tenure and Tenure Process Experiences of Canadian Nursing Faculty. Quality Advancement in Nursing Education - Avancées en formation infirmière.

[3] Newman KM (2017). Pre-tenures remain survival wise: How to survive your ?rst year in a tenure-track nursing faculty position. Journal of Nursing Education and Practice.

[4] Kulage KM, Schnall R, Hickey KT, Travers J, Zezulinski K, Torres F, Burgess J, Larson E (2015). Time and costs of preparing and submitting an NIH grant application at a school of nursing. Nursing Outlook.

[5] Smeltzer SC, Cantrell MA, Sharts-Hopko NC, Heverly MA, Jenkinson A, & Nthenge S (2016). Assessment of the impact of teaching demands on research productivity among doctoral nursing program faculty. Journal of Professional Nursing.

[6] (2017). Historical success rates in CIHR open grant competitions. Government of Canada.

[7] (2019). Funding: Success rates. National Institute of Health.

[8] (2018). Standard ARC schemes. Australian Research Council.

[9] (2019). UKRI Medical Research Council: Success rates. UK Research and Innovation.

[10] Tingen MS, Burnett AH, Murchison RB, Zhu H (2009). The importance of nursing research. Journal of Nursing Research.

[11] Cleary M, Sayers J, Watson R (2016). Essentials of building a career in nursing research. Nurse Researcher.

[12] Hess K, Steffes A (2011). What one takes for ?granted? about grant writing. Kansas Nurse.

[13] Freel SA, Smith PC, Burns EN, Downer JB, Brown AJ, Dewhirst MW (2017). Multidisciplinary mentoring programs to enhance junior faculty research grant success. Academic Medicine: Journal of the Association of American Medical Colleges.

[14] Brownson RC, Colditz GA, Dobbins M, Emmons KM, Kerner JF, Padek M, Proctor EK, Stange KC (2015). Concocting that magic elixiruccessful grant application writing in dissemination and implementation research. Clinical and Translational Science.

[15] Wisdom JP, Riley H, Myers N (2015). Recommendations for writing successful grant proposals: An information synthesis. Academic Medicine: Journal of the Association of American Medical Colleges.

[16] (2015). Thought experiments. Oxford Bibliographies.

[17] (2019). Think piece. Merriam-Webster.

[18] (2019). Thought experiments. Stanford Encyclopedia of Philosophy.

[19] Brown J (2006). The promise and perils of thought experiments. Interchange; 37(1-2).

[20] Brown Jr (2017). Counter Thought Experiments. Roy. Inst. Philos. Suppl.

[21] Brown Jr (1986). Thought experiments since the scientific revolution. International Studies in the Philosophy of Science.

[22] Sayama H (2015). Introduction to the modeling and analysis of complex systems.

[23] Barnes M, Hanson C, Giraud-Carrier C (2018). The case for computational health science. Journal of Healthcare Informatics Research.

[24] Hammond R, Osgood N, Wolfson M (2017). Using complex systems simulation modeling to understand health inequality. In Kaplan G, Diez Roux A, Simon C. Galea S (Eds.). Growing inequality: Bridging complex systems, population health and health disparities.

[25] Eckardt Patricia, Culley Joan M, Corwin Elizabeth, Richmond Therese, Dougherty Cynthia, Pickler Rita H, Krause-Parello Cheryl A, Roye Carol F, Rainbow Jessica G, DeVon Holli A (2017). National nursing science priorities: Creating a shared vision. Nurs Outlook.

[26] McLean A, McDonald W, Goodridge D, Osgood N (2019). Agent-based modeling: A method for investigating challenging research problems. Nursing Research.

[27] McKinnon T, Brunetto L, Teaford D, Meszaros M, O'Leary-Kelley C (2018). “The First Knock”: A Public Health Nursing Simulation. Clinical Simulation in Nursing.

[28] Hayden J, Smiley R, Alexander M, Kardong-Edgren S, Jeffries P (2014). The NCSBN National Simulation Study: A longitudinal, randomized, controlled study replacing clinical hours with simulation in prelicensure nursing education. Journal of Nursing Regulation.

[29] Smith PC, Hamilton BK (2015). The Effects of Virtual Reality Simulation as a Teaching Strategy for Skills Preparation in Nursing Students. Clinical Simulation in Nursing.

[30] Carlson KJ, Gagnon DJ (2016). Augmented Reality Integrated Simulation Education in Health Care. Clinical Simulation in Nursing.

[31] Garrett B, Taverner T, McDade P (2017). Virtual reality as an adjunct home therapy in chronic pain management: An exploratory study. JMIR Medical Informatics.

[32] Page S (2018). The model thinker: What you need to know to make data work for you.

[33] Borshchev A (2013). The big book of simulation modeling: Multimethod modeling with AnyLogic.

[34] Marshall Deborah A, Burgos-Liz Lina, IJzerman Maarten J, Crown William, Padula William V, Wong Peter K, Pasupathy Kalyan S, Higashi Mitchell K, Osgood Nathaniel D, ISPOR Emerging Good Practices Task Force (2015). Selecting a dynamic simulation modeling method for health care delivery research-part 2: report of the ISPOR Dynamic Simulation Modeling Emerging Good Practices Task Force. Value Health.

[35] Cerdá Magdalena, Keyes Katherine M (2019). Systems Modeling to Advance the Promise of Data Science in Epidemiology. Am J Epidemiol.

[36] Marshall Deborah A, Burgos-Liz Lina, IJzerman Maarten J, Osgood Nathaniel D, Padula William V, Higashi Mitchell K, Wong Peter K, Pasupathy Kalyan S, Crown William (2015). Applying dynamic simulation modeling methods in health care delivery research-the SIMULATE checklist: report of the ISPOR simulation modeling emerging good practices task force. Value Health.

[37] Skinner Adam, Walker Pippy, Atkinson Jo-An, Whitehead Rebecca, Roselli Tim, West Mark, Bright Margaret, Heffernan Mark, McDonnell Geoff, Veerman Lennert, Prodan Ante, Thomas David P, Burton Suzan (2019). Policy options for endgame planning in tobacco control: a simulation modelling study. Tob Control.

[38] Marshall B (2017). Agent-based modeling. In El-Sayed A. Galea S (Eds.). Systems science and population health.

[39] Wilensky U, Rand W (2015). An introduction to agent-based modeling: Modeling natural, social, and engineered complex systems with NetLogo.

[40] Macal CM (2017). Everything you need to know about agent-based modelling and simulation. Journal of Simulation.

[41] Railsback S, Grimm V (2012). Agent-based and individual-based modeling: A practical introduction.

[42] Nianogo Roch A, Arah Onyebuchi A (2015). Agent-based modeling of noncommunicable diseases: a systematic review. Am J Public Health.

[43] Badham Jennifer, Chattoe-Brown Edmund, Gilbert Nigel, Chalabi Zaid, Kee Frank, Hunter Ruth F (2018). Developing agent-based models of complex health behaviour. Health Place.

[44] Glover Matthew J, Jones Edmund, Masconi Katya L, Sweeting Michael J, Thompson Simon G, SWAN Collaborators, SWAN collaborative group (2018). Discrete Event Simulation for Decision Modeling in Health Care: Lessons from Abdominal Aortic Aneurysm Screening. Med Decis Making.

[45] Siddiqui S, Morse E, Levin S (2017). Evaluating nurse staffing levels in perianesthesia care units using discrete event simulation. IISE Transactions on Healthcare Systems Engineering.

[46] AnyLogic C AnyLogic simulation software. 2020.

[47] International CON 2020: International Year of the Nurse and Midwife. 2020.

[48] Thorne S (2019). Nursing now or never. Nurs Inq.

